# You Are What You Eat: A Review on Dietary Interventions for Treating Pediatric Nonalcoholic Fatty Liver Disease

**DOI:** 10.3390/nu15153350

**Published:** 2023-07-28

**Authors:** Piper Sandel, Lawrence Ma, Helen Wang, Eric A. Pasman

**Affiliations:** 1 Section of Academic General Pediatrics, Department of Pediatrics, University of California San Diego, San Diego, CA 92123, USA; lma@health.ucsd.edu (L.M.); hew004@health.ucsd.edu (H.W.); 2 Division of Pediatric Gastroenterology, Department of Pediatrics, Naval Medical Center San Diego, San Diego, CA 92134, USA; eric.a.pasman.mil@health.mil; 3 Department of Pediatrics, Uniformed Services University of the Health Sciences, Bethesda, MD 20814, USA

**Keywords:** pediatric nonalcoholic fatty liver, dietary interventions, nutrition

## Abstract

As the obesity pandemic worsens, cases of pediatric nonalcoholic fatty liver disease (NAFLD) and complications of this disease, such as progressive liver failure, in young adults will continue to rise. Lifestyle changes in the form of dietary modifications and exercise are currently first-line treatments. Large pediatric-specific randomized controlled trials to support specific interventions are currently lacking. A variety of dietary modifications in children with NAFLD have been suggested and studied with mixed results, including low-sugar and high-protein diets, the Mediterranean diet, and the Dietary Approach to Stop Hypertension (DASH). The roles of dietary supplements such as Vitamin E, polyunsaturated fatty acids (PUFAs), ginger, and probiotics have also been investigated. A further understanding of specific dietary interventions and supplements is needed to provide both generalizable and sustainable dietary recommendations to reverse the progression of NAFLD in the pediatric population.

## 1. Introduction

Nonalcoholic fatty liver disease (NAFLD) is the most common liver disease in children, and the burden of this disease is growing [1,2]. Its estimated prevalence in children is around 10% in the general population and up to 34% in obese children [1,3]. It is more common in boys than girls, and more common in Hispanic children, followed by Asian children, compared to non-Hispanic black and white children [1,3]. There is a lack of long-term outcome studies evaluating pediatric NAFLD patients; however, data show that like adult NAFLD, it can progress to severe disease, including cirrhosis and end-stage liver disease [4]. Pediatric onset NAFLD may in fact be more severe than adult onset NAFLD given the earlier age of disease development [5]. The progression of adult NAFLD is projected to become the most common reason for liver transplant in the United States, which highlights the importance of addressing and reducing NAFLD before adulthood [6,7]. 

NAFLD represents a broad histologic spectrum of disease that includes nonalcoholic fatty liver, nonalcoholic steatohepatitis (NASH), and the more severe liver fibrosis and cirrhosis. It is characterized by the accumulation of hepatic fat secondary to excessive peripheral lipolysis and hepatic de novo lipogenesis (DNL) in the absence of excess alcohol consumption [8,9]. NAFLD pathophysiology is complex and involves a multifactorial interplay of genetic, hormonal, nutritional, and environmental factors [10]. Pediatric NAFLD is associated with insulin resistance and obesity, with common comorbidities including type 2 diabetes, hypertension, and dyslipidemia [11]. 

Children with NAFLD are often asymptomatic; thus, screening for this chronic disease is imperative to improve outcomes through early detection and intervention [11]. The North American Society For Pediatric Gastroenterology, Hepatology & Nutrition (NASPGHAN) made recommendations in 2016 on the diagnosis and management of pediatric NAFLD, and the screening guidelines are endorsed by the American Academy of Pediatrics [11,12]. NASPGHAN recommends using serum alanine transaminase (ALT) to screen children, starting at the age of 9–11 years, who are obese or who are overweight, with additional risk factors including central adiposity, insulin resistance, pre-diabetes or diabetes, dyslipidemia, sleep apnea, or a family history of NAFLD. Those children who have persistently elevated ALT levels, defined as being above 22 mg/dl for girls and 26 mg/dl for boys, need further investigation. As NAFLD is a diagnosis of exclusion, other differential diagnoses such as viral hepatitis, autoimmune disease, or exposure to hepatotoxic medications need to be ruled out first. Children who are overweight or obese with two times the sex-specific ALT normal value and no other likely cause of liver disease have a high likelihood of NAFLD [11]. It is important to note that the level of elevation of ALT does not reliably predict the level of severity of NAFLD [13]. The gold standard for NAFLD diagnosis requires a liver biopsy with histologic confirmation of at least 5% hepatic steatosis [11]. 

The treatment goal for pediatric NAFLD is to decrease liver steatosis and inflammation. ALT values are often used as a stand-in marker to assess the response to treatment, given that repeated liver biopsies are not practical in the pediatric population [11]. There are no available medications to treat NAFLD. The first-line treatments for NAFLD include lifestyle modifications through dietary changes and exercise, given the strong association of NAFLD with obesity. However, the specific type of diet to recommend is still unclear. A variety of dietary modifications in children with NAFLD have been suggested and studied with mixed results, including low-sugar and high-protein diets, the Mediterranean diet, DASH, and adding dietary supplements. The following is a review of the current research on these diets and supplements.

## 2. Dietary Treatment

### 2.1. Low Sugar

A high intake of dietary added sugars, particularly fructose, is linked to the development and severity of NAFLD [14,15]. Though the mechanism is not fully understood, fructose directly stimulates DNL, which, in turn, leads to hepatic lipid accumulation [8]. Many children consume excessive amounts of added sugars, primarily in the form of sugar-sweetened beverages (SSB). Therefore, dietary interventions reducing the intake of added sugars may prove promising for the treatment of NAFLD [16]. 

Mager et al. (2015) examined plasma markers of liver dysfunction after a dietary intervention to reduce fructose consumption. The enrolled participants (12 children with NAFLD and 14 healthy controls) received dietary education and received sample menus and strategies to reduce their glycemic index, glycemic load, and fructose, but prepared and chose their own food within the guidelines. After six months of dietary fructose restriction, the children with NAFLD had decreased plasma levels of ALT [17].

Schwarz et al. (2017) conducted a non-controlled study examining the impact of fructose restriction among 41 Latino and African American children aged 9–18 who were obese and routinely consumed >50 g per day of sugar, over a 9-day period. Participants consumed diets in which they received isocaloric substitution of starches for most sugar. Fructose was restricted to 4% of participants’ daily energy intake. On day 10, they saw a decrease in hepatic fat, DNL, and visceral fat using magnetic resonance spectroscopy and imaging. This suggests that fructose intake, regardless of the total daily calories consumed, is associated with NAFLD. This work supports the idea that fructose restriction is an important step in the treatment and prevention of NAFLD [8].

This was further demonstrated by Schwimmer et al. (2019) in a randomized clinical trial that investigated the impact of a low-sugar diet on NAFLD. The study included 40 adolescent boys, the majority of whom were Hispanic, aged 11 to 16 years with NAFLD, who were randomized into either a usual diet group or a low-sugar diet group (with free sugar comprising less than 3% of their total daily calories). At baseline, these participants regularly consumed SSB. Participants randomized into the low-sugar diet group received and ate food purchased and provided by the study team. After eight weeks, researchers found that a diet low in free sugars compared with usual diet resulted in a greater reduction in hepatic steatosis, measured via magnetic resonance imaging (MRI) of the proton density fat fraction [18]. A subset of 29 of the study participants completed a protocol using stable isotope tracers, allowing for the change in hepatic DNL to be measured. The researchers found that there was a significant reduction in hepatic DNL in the low-sugar diet group as compared to the usual diet group [19].

Goss et al. (2020) compared restrictions in dietary carbohydrates and dietary fat in 32 children with obesity and NAFLD over an 8-week period. In this randomized controlled trial, families were provided with meals for two weeks, and then, prepared meals under specified instructions for the final six weeks. Hepatic lipid content decreased more in the carbohydrate-restricted group [20]. 

Many children consume well over the recommended amounts of added sugars in their diets. Early limited research suggests diets that restrict fructose lead to improvements in NAFLD. A low-sugar diet is a targetable goal and may best be achieved through SSB reduction [19]. This is particularly important, as Jin et al. (2014) found that reduced fructose consumption may slow atherosclerosis progression in NAFLD patients [21]. In their four-week randomized controlled double-blinded trial, 24 Hispanic American adolescents, who were usual consumers of at least three servings of SSB per day, were randomized to receive three servings of calorie-matched fructose-only or glucose-only beverages per day. At four weeks, there was no change in hepatic fat or body weight; however, the group randomized to the glucose-only beverages had significant improvement in factors related to cardiovascular disease progression: insulin sensitivity, high-sensitivity C-reactive protein (hsCRP), and low-density lipoprotein (LDL) oxidation. Though longer-term studies that evaluate the addition of other dietary sources of fructose in a broader population are needed, their study suggests that reducing fructose intake in patients with NAFLD may have health benefits beyond just the liver. 

### 2.2. Dietary Approach to Stop Hypertension (DASH)

The DASH diet, developed in 1997, emphasizes an increased intake of whole grains, low-fat dairy, low-fat poultry meat, legumes, fish, vegetables, and fruits, and a reduced intake of cholesterol and total and saturated fats [22]. The increased fiber and mineral (especially calcium, potassium, and magnesium) intake coupled with the decreased consumption of simple sugars and saturated fats is postulated to reduce inflammation and oxidative stress [23]. Meta-analyses of the DASH diet demonstrate its effectiveness in inducing weight loss, decreasing body mass index (BMI), and decreasing waist circumference in adults [24]. All of these are potential benefits of the DASH diet that could be applied to the treatment of NAFLD. Currently, one trial in adults (2016) and none in children have studied using the DASH diet to treat patients with NAFLD. This 8-week trial randomly assigned adult patients who were overweight or obese and who had NAFLD to either a control diet or the DASH diet. Patients randomized to the DASH diet demonstrated significant reductions in weight, waist circumference, serum ALT, triglycerides, and hsCRP compared to patients randomized to the control diet. More importantly, 80% of the patients in the DASH diet group demonstrated a decreased grade of NAFLD on ultrasound imaging compared to 40% of the patients in the control diet group [25]. Though there are no studies that evaluate the DASH diet in pediatric patients with NAFLD, the DASH diet has been proven to reduce inflammation, weight, and risk of developing diabetes in pediatric patients with obesity and metabolic syndrome [26,27,28]. These studies provide indirect evidence that the DASH diet may be beneficial for pediatric patients with NAFLD, given the similar alterations in the metabolic pathways underlying NAFLD, metabolic syndrome, and insulin resistance. 

### 2.3. Mediterranean Diet (MD)

The MD is a dietary pattern characterized by a high intake of unrefined whole grains, vegetables, fruits, nuts, fish, legumes, and olive oil combined with moderate amounts of dairy and low consumption of red meat. There is a high intake of foods rich in complex carbohydrates, fiber, antioxidants, phytochemicals, and both monounsaturated fatty acids (MUFAs) and PUFAs [29,30]. This dietary pattern is believed to offer antioxidant and anti-inflammatory benefits while also positively impacting the biodiversity of the gut microbiome [31,32]. The higher levels of MUFAs and PUFAs and lower levels of saturated fatty acids results in reduced hepatic lipogenesis and inflammation, decreased insulin resistance, and changes in the distribution of visceral fat deposition [31,32,33]. Thus, the MD is promising as a potential treatment for patients with NAFLD. A meta-analysis (2022) assessing the MD as an intervention in adult patients with NAFLD showed significant improvements in ALT, fatty liver index, hepatic steatosis, and liver stiffness measurements, even in the absence of significant weight loss [34]. There is currently only one longitudinal clinical trial studying the MD as an intervention in pediatric patients with NAFLD. This study (2022) randomized children aged 9–17 years who were overweight and had NAFLD to a 12-week dietary intervention with either a Mediterranean or a low-fat diet. Of note, participants in both groups were mildly calorie-restricted and participated in an exercise program. The primary outcomes included changes in hepatic steatosis and ALT levels, with the secondary outcome evaluating insulin resistance. At the end of the intervention, both groups had similar decreases in measurements of hepatic steatosis and liver stiffness, and all participants’ ALT levels normalized. However, children in the MD intervention group demonstrated more significant reductions in a measure of insulin resistance. The study authors concluded that both diets appeared effective for improving hepatitis steatosis and normalizing ALT levels, but children in the MD arm were able to achieve these results with less overall caloric restriction [35].

### 2.4. High-Protein Diet 

High-protein diets may be advantageous for those who need to achieve weight loss. Diets that favor a high intake of protein over carbohydrate intake have been shown to reduce weight and improve high-density lipoprotein (HDL) levels in adults [36]. There is limited knowledge of the role of proteins in the development or progression of NAFLD [37]. A study in animals has shown that soy protein in the diet can lower lipid levels in the blood and liver [38]. Additional evidence suggests that a high-protein diet could be beneficial in the treatment of NAFLD as the increased amino acid catabolism leads to an increase in hepatic energy expenditure through increased hepatic lipid oxidation [39]. However, Western-style high-protein diets that are high in processed and red meats increase the risk of heart disease and are associated with increased NAFLD prevalence [40]. There are limited studies in the adult population and no studies in the pediatric population examining the effects of a high-protein diet on NAFLD. 

Bezerra Duarte et al. (2014) examined the impact of a low-calorie high-protein diet over a 78-day period on 48 adult participants with NAFLD. The prescribed diet contained 35% protein, both animal and vegetable, with an allotment of 1200 calories per day for female participants and 1400 calories per day for male participants. They found that total cholesterol, LDL, triglycerides, aspartate aminotransferase (AST), gamma-glutamyl transpeptidase (GGT), alkaline phosphatase (AP), Hemoglobin A1c (HgbA1c), and fasting blood glucose levels significantly decreased, while BMI remained unchanged. The generalizability of these results is limited by the short duration of the study [41]. Additionally, in adults with both type 2 diabetes and NAFLD, Markova et al. (2017) found reductions in intrahepatic lipids with isocaloric high-protein diets containing either animal or plant protein over a 6-week period [42].

There remains controversy regarding the impact of high-protein diets on liver health. Further studies need to be conducted to elucidate whether a high-protein diet should be recommended for the treatment of NAFLD and the impact of animal protein versus plant-based protein. More importantly, long-term pediatric-specific studies are needed to determine sustainability and for any negative impacts of high-protein diets.

### 2.5. Supplements

Multiple dietary supplements have been examined as potential treatments in pediatric NAFLD. Recent research focusing on antioxidants, PUFAs, and probiotics is summarized below. 

### 2.6. Antioxidants

Obese children are known to have decreased serum levels of the antioxidant alpha-tocopherol, which increases their risk for oxidative stress and likely contributes to NAFLD pathogenesis [43]. Multiple studies have looked at the role of supplementation with antioxidants like Vitamin E as a potential treatment for NAFLD. 

A study by Wang et al. (2008) compared the impact of Vitamin E supplementation and lifestyle interventions among obese Chinese children with NAFLD. Over a one-month period, 76 participants aged 10–17 years were divided into a control group, an active lifestyle intervention group, and a supplementation group. Serum markers and BMI were examined at baseline and after one month of the intervention. Both intervention groups showed improvements in ALT and BMI, while the control group did not; improvements were more significant in the lifestyle intervention group [44]. In another study by Nobili et al. (2008), 53 patients aged 5–18 years underwent a lifestyle intervention with or without Vitamin E and C supplementation over a 24-month period. Lifestyle interventions included a calorie-specific diet and increased physical activity. Patients were randomized to receive either a placebo or 600 international units (IU)/day Vitamin E and 500 mg/day Vitamin C supplements. Both groups demonstrated weight loss and improvement in liver histology and ALT levels, but the results between the two groups did not differ significantly. This work suggests that Vitamin E with Vitamin C does not increase the efficacy of lifestyle interventions [45]. The effects of Vitamin E were further studied by Lavine et al. (2011). In their randomized, double-blind, double-dummy, placebo-controlled study, 173 participants aged 8–17 years with NAFLD, were given Vitamin E, metformin, or a placebo over a 96-week treatment period. ALT levels were examined at set periods and at 24 weeks post-treatment cessation. Neither Vitamin E nor metformin was shown to be superior to the placebo in sustaining a reduction in ALT. However, among a subset of patients with NASH, Vitamin E led to a significantly greater resolution of NASH as compared to the placebo [46]. Overall, the benefit of Vitamin E supplementation is unclear, particularly in patients with NAFLD but not NASH, especially in light of ongoing safety concerns with long-term use [47]. 

Ginger is another antioxidant with anti-inflammatory properties that may have promise as an adjunct in the treatment of pediatric NAFLD. Kamari et al. (2023) conducted a randomized controlled trial on 160 obese children between the ages of 8 and 11 years examining the impact of 100 mg ginger supplements in conjunction with an anti-inflammatory diet low in processed foods. Children were divided into four groups: ginger supplement, ginger supplement with the anti-inflammatory diet, control, and anti-inflammatory diet alone. Hepatic steatosis, measured via ultrasound and ALT levels, decreased in the ginger and ginger-with-anti-inflammatory-diet groups [48]. 

### 2.7. Polyunsaturated Fatty Acids

NAFLD is associated with an increase in hepatic saturated fatty acids and MUFAs and a decrease in PUFAs [49,50]. Dietary PUFAs, from sources such as walnuts, flaxseed, and fish, are known to inhibit hepatic DNL [51]. Supplementation with PUFAs, including docosahexaenoic acid (DHA) and eicosapentaenoic acid (EPA), has been examined as a potential treatment for NAFLD. A randomized controlled study by Nobili et al. (2011–2013) examined the impact of DHA supplementation versus a placebo on liver fat content in 60 children with NAFLD. Children received either supplementation with 250 mg DHA, 500 mg DHA, or a placebo over a 6–24-month period. Ultrasonographic measurements of liver fat content showed improved liver steatosis in children supplemented with DHA as compared to the placebo group; there was no significant difference between the two doses of DHA [52,53]. Pacifico et al. (2015) also found that hepatic fat, as measured via MRI, decreased over a 6-month period in their DHA intervention group compared to their control group. In their double-blind, placebo-controlled randomized trial, 58 children with NAFLD were either supplemented with 250 mg DHA supplementation or a placebo [54].

Janczyk et al. (2015) examined DHA supplementation with EPA supplementation. In their 6-month-long study, children aged 11–15 years who were overweight and/or obese and had NAFLD were either supplemented with DHA and EPA supplement ranging from 450 mg–1300 mg/day depending on patient weight, or a placebo (omega-6 sunflower oil). There was no difference in hepatic steatosis on ultrasound or ALT levels between the intervention group and placebo groups. However, they did find an improvement in GGT levels and AST levels among the treatment group [55]. A study by Boyraz et al. (2015) also investigated the impact of 1000 mg daily EPA/DHA supplementation against a placebo, but both groups also underwent a lifestyle intervention. They enrolled 108 children aged 8–17 years with obesity and NAFLD and trended their ALT over a 12-month period. At the end of the study period, both groups had a decrease in ALT [56]. A study by Spahis et al. (2018) also found that 6 months of 2 g daily n-3 PUFA supplementation in 20 individuals with NAFLD led to a reduction in ALT levels [57]. These studies suggest there could be a benefit of PUFA supplementation, but the optimal type and dosage of the PUFA and treatment duration remain to be clarified. 

### 2.8. Probiotics 

As the liver receives 70% of its blood supply directly from the intestines via the portal vein, alterations in the intestinal microbiome can result in increased hepatic exposure to bacteria-derived lipopolysaccharides and endotoxins. These molecules contribute to the pathogenesis of NAFLD via increased inflammation, oxidative stress, and intrahepatic fat accumulation [58]. Thus, using probiotics to alter the microbiome could have beneficial effects on liver health. Famouri et al. (2017) demonstrated improvements in ALT levels and an increased likelihood of normalized ultrasound findings in obese children with NAFLD treated with 12 weeks of a daily probiotic (a blend of *Lactobacillus acidophilus, Lactobacillus rhamnosus, Bifidobacterium lactis,* and *Bifidobacterium bifidum*) compared to those treated with a placebo [59]. Vajro et al. (2011) also noted reductions in ALT levels in obese children with NAFLD who were treated for 8 weeks with a daily probiotic (*Lactobacillus rhamnosus* strain GG) compared to those treated with a placebo [60]. Alisi et al. (2014) documented improvements in liver ultrasound findings in children with biopsy-confirmed NAFLD after a 12-week course of VSL#3 (a blend of eight strains of bacteria) [61]. Although these studies are promising, their limitations include small sample sizes, short durations of follow-ups, and variability in the probiotic strains used, which makes generalizability challenging. Pending large-scale randomized clinical trials, there is currently no recommendation to use probiotics to treat NAFLD.

Evidence remains mixed on dietary supplements as a treatment for pediatric NAFLD, and more research is needed before recommendations can be made.

## 3. Conclusions

NAFLD is the most common form of pediatric chronic liver disease, with prevalence estimates of upwards of 30% in obese children [3]. With the rising rates of childhood obesity across the world and the potential for NAFLD to progress to fibrosis and end-stage liver disease, a significant rise in NAFLD-associated morbidity and mortality is expected to follow [62]. Currently, there are no proven pharmacological interventions for treating or reversing NAFLD. Management recommendations from major organizations, including the Expert Committee on NAFLD and NASPGHAN, focus on lifestyle interventions, but no specific dietary intervention is recommended [11]. In adult patients, 5–10% weight loss has been shown to result in the regression of steatosis and fibrosis [63], but the exact amount of weight loss needed in pediatric patients is less clear, and there are other potential long-term health consequences of significant caloric restriction. This article reviewed the existing literature on specific dietary interventions and nutritional supplements that may improve NAFLD in children. Limiting sugary beverages and added fructose in the diet appears to be a promising intervention due to the clear biochemical pathways linking fructose to DNL. Trials have demonstrated that reduced fructose intake results in decreased ALT levels and hepatic fat on imaging. Studies in pediatric patients looking at the DASH and the MD are encouraging but limited by small sample sizes and short durations of follow-ups. Dietary supplements including PUFAs, nutraceuticals, and probiotics may prove to be promising in the treatment of NAFLD. Ultimately, further research with large and diverse populations of pediatric patients is needed to identify what specific dietary interventions and supplements are most effective, with particular emphasis on safety, generalizability, and sustainability. In the meantime, emphasis on preventing childhood obesity, and hence, the development of NAFLD through healthier eating remains critical.

## Data Availability

Not applicable.
